# Adverse Outcome Pathway 298: Increase in Reactive Oxygen Species Leading to Human Treatment-Resistant Gastric Cancer

**DOI:** 10.3390/cancers18020268

**Published:** 2026-01-15

**Authors:** Shihori Tanabe, Sabina Quader, Ryuichi Ono, Horacio Cabral, Edward J. Perkins

**Affiliations:** 1 Division of Risk Assessment, Center for Biological Safety and Research, National Institute of Health Sciences, 3-25-26, Tonomachi, Kawasaki-ku, Kawasaki 210-9501, Japan; 2 Innovation Centre of NanoMedicine (iCONM), Kawasaki Institute of Industrial Promotion, Kawasaki 210-0821, Japan; 3 Division of Cellular and Molecular Toxicology, Center for Biological Safety and Research, National Institute of Health Sciences, Kawasaki 210-9501, Japan; 4 Department of Bioengineering, Graduate School of Engineering, The University of Tokyo, Tokyo 113-0033, Japan; 5 Environmental Laboratory, US Army Engineer Research and Development Center, Vicksburg, MS 39180, USA

**Keywords:** adverse outcome pathway (AOP), epithelial–mesenchymal transition (EMT), gastric cancer, reactive oxygen species (ROS), Wnt signaling

## Abstract

Injury causes resistance in human gastric cancer. Adverse Outcome Pathway (AOP) 298, entitled “increase in reactive oxygen species (ROS) leading to human treatment-resistant gastric cancer,” consists of “increase in ROS” as a molecular initiating event (MIE), followed by a series of key events (KEs), namely “porcupine-induced Wnt secretion and Wnt signaling activation,” “beta-catenin activation,” and “epithelial–mesenchymal transition (EMT),” and the adverse outcome (AO) of “treatment-resistant gastric cancer” in the sequence. AOP 298 includes four KE relationships (KERs): “increase in ROS leads to porcupine-induced Wnt secretion and Wnt signaling activation,” “porcupine-induced Wnt secretion and Wnt signaling activation leads to beta-catenin activation,” “beta-catenin activation leads to EMT,” and “EMT leads to treatment-resistant gastric cancer.” ROS has multiple roles in disease, such as in the development and progression of cancer, or apoptotic induction, causing anti-tumor effects. Regarding AOP 298, we focus on the role of sustained chronic ROS levels in inducing therapy resistance in human gastric cancer. EMT, induced by Wnt/beta-catenin signaling, demonstrates cancer stem cell-like characteristics in human gastric cancer.

## Simple Summary

This research aims to elucidate a pathway starting with increases in reactive oxygen species (ROS) and leading to human treatment-resistant gastric cancer through Wnt/beta-catenin signaling and epithelial–mesenchymal transition (EMT). The main topic in this article is Adverse Outcome Pathway (AOP) 298, entitled “increase in reactive oxygen species (ROS) leading to human treatment-resistant gastric cancer.” It consists of a molecular initiating event (MIE), “increase in ROS”; three key events (KEs), namely “porcupine-induced Wnt secretion and Wnt signaling activation,” “beta-catenin activation,” and “epithelial–mesenchymal transition (EMT)”; and an adverse outcome (AO), “treatment-resistant gastric cancer,” illustrating a mechanism of human treatment-resistant gastric cancer induced by drugs, therapy, or radiation.

## 1. Introduction

Molecular signaling pathway networks are regulated in epithelial–mesenchymal transition (EMT) and cancer stem cells (CSCs), which exhibit anti-cancer drug-resistant features. The NRF2-mediated oxidative stress response network included molecules related to EMT regulation through the growth factor pathway and the production of nitric oxide and reactive oxygen species in macrophages such as PI3K and AKT [1,2,3]. NRF2 signaling regulated EMT in gastric cancer [4]. Additionally, EMT induction increased metastasis and cisplatin resistance in gastric cancer, which involved Nrf2 signaling [5].

This research aims to ensure the safety of therapeutics such as anti-cancer drugs by revealing the molecular mechanisms that contribute to their efficacy and side effects or unexpected and off-targeted adverse effects. Chemicals induce molecular alterations and body responses. Recent progress in cellular and molecular network pathway analysis has revealed the activation mechanisms of cellular signal transduction upon cancer and chemical stimulation. In developing anti-cancer drugs such as molecular-targeting therapeutics, identifying target molecules and inhibiting or activating the signaling transduction related to the target molecules is important. Anti-cancer therapeutics targeting the Wnt/beta-catenin signaling pathway regulating cell self-renewal, the Hedgehog signaling pathway, Notch signaling pathway, and EGFR receptor signaling pathway have been developed and approved; however, off-target effects for molecular network pathways are not fully understood. To elucidate the safety of molecular-targeted and cellular therapeutics using multipotent stem cells, it is critical to predict unexpected off-target network pathways. Molecular network pathway analysis utilizing the existing abundant data in databases is needed [6,7]. This study aims to predict the side effects or adverse effects of different therapeutics by analyzing the molecular network pathway dynamism utilizing data from databases.

**Figure 1:**
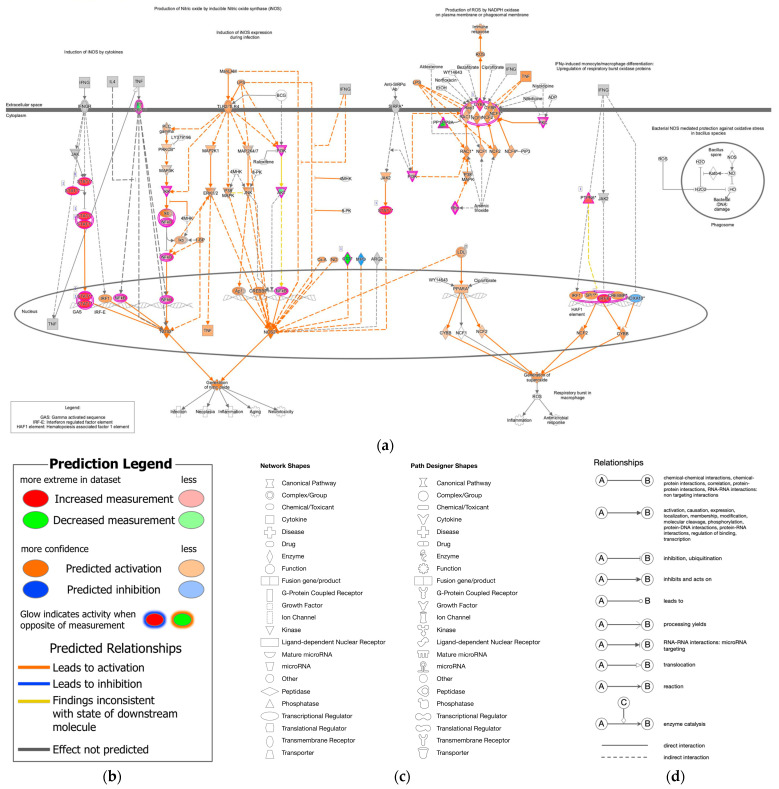
Canonical pathway of nitric oxide and reactive oxygen species production in macrophages and the molecular relation to epithelial–mesenchymal transition (Ingenuity Pathway Analysis (IPA)). (**a**) The production of nitric oxide and reactive oxygen species in macrophages was overlaid with analysis of 12-stomach cancer 14,264 (As of June 2023). The superoxide was predicted to be generated in the stomach cancer. (**b**) The prediction legend of the pathway is shown. Red or green coloring indicates upregulated or downregulated gene expression, respectively. Orange or blue coloring indicates predicted activation or inhibition, respectively. The intensity of the colors indicates the degree of up- or down-regulation. An orange or blue line indicates activation or inactivation, respectively. (**c**) The legend for the node shapes in the pathway is shown. (**d**) The legend for the relationship lines is also shown. Gene/Protein/Chemical identifies marked with an asterisk (*) indicate that multiple identifies in the dataset file map to a single gene/chemical in the Global Molecular Network in IPA.

ROS consist of free oxygen radicals, such as superoxide, hydroxyl radical, nitric oxide, organic radicals, peroxyl radicals, alkoxyl radicals, thiyl radicals, sulfonyl radicals, thiyl peroxyl radicals, and disulfides, as well as non-radical ROS such as hydrogen peroxide, singlet oxygen, ozone/trioxygen, organic hydroperoxides, hypochlorite, peroxynitrite, nitrosoperoxycarbonate anion, nitrocarbonate anion, dinitrogen dioxide, nitronium, and highly reactive lipid- or carbohydrate-derived carbonyl compounds [8]. ROS have double-edged effects, which may affect tumorigenesis. ROS play crucial roles in protecting humans from infection, whereas prolonged excess ROS cause several diseases, including cancer, sensory impairment, and cardiovascular, neurological, and psychiatric diseases [9]. Nicotinamide adenine diphosphate (NADPH) oxidase catalyzes the production of superoxide through the one-electron reduction of oxygen and produces ROS [10] (Figure 1).

## 2. Outline of AOP298

### 2.1. Structure of AOP298

**Figure 2:**
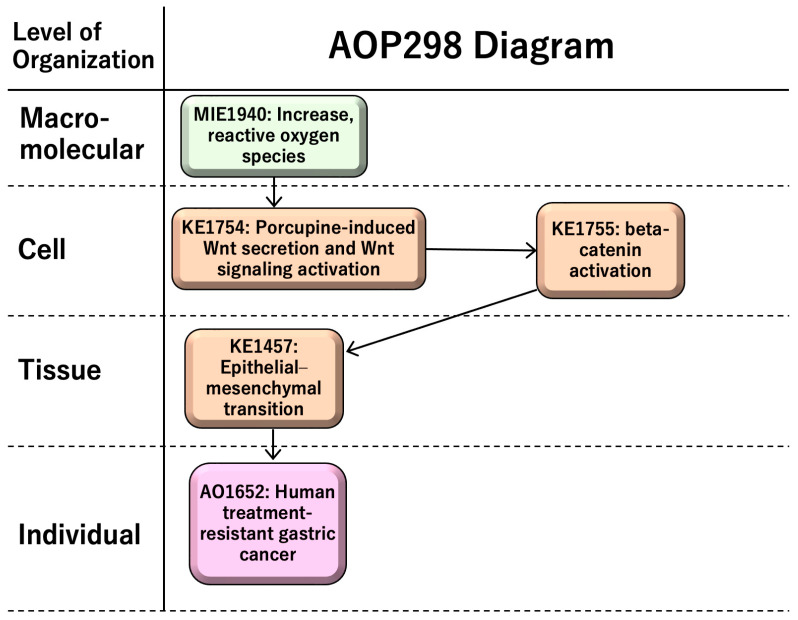
AOP 298: “increase in ROS leading to treatment-resistant gastric cancer”.

AOP 298, entitled “increase in reactive oxygen species (ROS) leading to human treatment-resistant gastric cancer,” consists of a molecular initiating event (MIE1; KE1115), an increase in ROS; key events (KEs), namely porcupine-induced Wnt secretion and Wnt signaling activation (KE1; KE1754), beta-catenin activation (KE2; KE1755), and epithelial–mesenchymal transition (EMT) (KE3; KE1457); and an adverse outcome (AO; KE1651)—namely, treatment-resistant gastric cancer (Figure 2). AOP 298 includes four KE relationships (KERs): “increase in ROS leads to porcupine-induced Wnt secretion and Wnt signaling activation,” “porcupine-induced Wnt secretion and Wnt signaling activation leads to beta-catenin activation,” “beta-catenin activation leads to EMT,” and “EMT leads to treatment-resistant gastric cancer” (https://aopwiki.org/aopwiki/snapshot/pdf_file/298-2025-08-15T02:01:48+00:00.pdf) (accessed on 4 December 2025) (Supplementary Material S1).

ROS have both benefits and risks for human health; chronic ROS, which is prolonged excess ROS, induces sustained tissue damage and macrophage activation. Porcupine-induced Wnt secretion in macrophages induces proliferation and beta-catenin activation, leading to epithelial–mesenchymal transition (EMT). EMT induces cancer migration and drug resistance, causing human treatment-resistant gastric cancer. AOP298-related information is summarized in Table 1.

### 2.2. Summary of Scientific Evidence Assessment

#### 2.2.1. MIE1; KE1115: Increase in Reactive Oxygen Species (ROS)

Increases in ROS are observed when cells are exposed to various stressors such as allergens, ionizing radiation, and chemicals [11]. ROS include free radicals (e.g., superoxide anion, hydroxyl radicals, nitric oxide, nitrogen dioxide, organic radicals, peroxyl radicals, alkoxyl radicals, thiyl radicals, sulfonyl radicals, thiyl peroxyl radicals, and disulfide) and non-radical ROS (hydrogen peroxide, singlet oxygen, ozone/trioxygen, organic hydroperoxides, hypochloride, peroxynitrite, nitrosoperoxycarbonate anion, nitrocarbonate anion, dinitrogen dioxide, nitronium, and highly reactive lipid- or carbohydrate-derived carbonyl compounds). Increases in ROS contribute to various diseases.

#### 2.2.2. KE1; KE1754: Porcupine-Induced Wnt Secretion and Wnt Signaling Activation

Sustained tissue damage induces inflammation. Wnt/beta-catenin signaling is essential for intestinal homeostasis, where macrophage-derived Wnt in intestinal repair is crucial for rescuing intestinal stem cells from radiation lethality [9].

#### 2.2.3. KE2; KE1755: Beta-Catenin Activation

The oncoprotein beta-catenin stabilizes and translocates to the nucleus, followed by induction of the ZEB1 transcription factor, which promotes epithelial–mesenchymal transition (EMT) [12]. One of the important signaling pathways inducing EMT is the canonical Wnt/beta-catenin pathway, where beta-catenin acts as a coactivator of T-cell and lymphoid enhancer (TCF-LEF) factors [13]. Beta-catenin/TCF4 binds to the ZEB1 promoter and induces transcription, leading to EMT, a main hallmark of malignant cells [12].

#### 2.2.4. KE3; KE1457: Epithelial–Mesenchymal Transition (EMT)

It is known that EMT plays an important role in therapeutic resistance and drug responses in human gastric cancer [7,14,15,16]. EMT is a critical regulator of the CSC phenotype and drug resistance [14] and is involved in the metastasis of gastric cancer [17,18]. Triggering receptor expressed on myeloid cells 2 (TREM2)—a key gene in gastric cancer progression—promotes EMT [19].

#### 2.2.5. AO; KE1651: Treatment-Resistant Gastric Cancer

Gastric cancer can be classified as diffuse- or intestinal-type with an mRNA ratio of CDH2 to CDH1 [20]. Diffuse-type gastric cancer, which has a poor prognosis and is treatment-resistant, has up-regulated genes that are involved in EMT [21,22]. Gastric cancer-derived mesenchymal stromal cell-primed macrophages promote metastasis and EMT in gastric cancer [23].

Scientific evidence of AOP298 is provided as support for the biological plausibility of KERs (Table 2), for the essentiality of KEs (Table 3), and as empirical support for KERs (Table 4).

## 3. Discussion

AOP298, entitled “increase in ROS leading to treatment-resistant gastric cancer,” consists of several components: “increase in ROS” as an MIE; “Porcupine-induced Wnt secretion and Wnt signaling activation,” “beta-catenin activation,” and “epithelial–mesenchymal transition (EMT)” as intermediate KEs; and “treatment-resistant gastric cancer” as an AO. The AOP’s description is based on a mechanism of drug resistance, metastasis, and gastric cancer progression, and its application involves the risk assessment of anti-cancer drugs and the development of anti-cancer treatment.

Chronic low-level increased ROS play crucial roles in the development of radioresistant gastric cancer via tumor microenvironment alteration and EMT [35]. Specific cellular adaptations—e.g., up-regulation of antioxidant systems such as Nrf2 or changes in mitochondrial function—may maintain the chronic state. Radiation promotes the metastasis of cancer via ROS and EMT [50]. The extent of ROS levels appears to be critical in balancing cancer cell growth and cell death [51]. Oxidative stress is linked to numerous inflammatory diseases and cancer, where oxidative stress and inflammation drive tumor cell proliferation, migration, invasion, and metastasis [52].

The tumor microenvironment (TME), consisting of immune cells, natural killer cells, the extracellular matrix, etc., plays a critical role in tumor initiation, development, and metastasis by manipulating redox signaling [53,54]. Interactions among tumor-associated macrophages, gastric cancer cells, and natural killer cells induce immune checkpoint molecules that interact with immune cells in the TME of gastric cancer, thereby evading anti-tumor immunity. [55]. Cancer cells use chronic ROS to neutralize, exhaust, and suppress anti-tumor immune cells [53]. Persistent oxidative stress signals from cancer cells transform fibroblasts into pro-tumorigenic cancer-associated fibroblasts [53]. Chronic ROS is crucial for activating the TME in treatment-resistant cancer.

There is a possibility that non-canonical Wnt signaling, independent of beta-catenin, is involved in EMT in prostate cancer [56]. Non-canonical Wnt signatures, such as ROR2 and FZD7, are correlated with poor prognosis in gastric cancer [57]. The involvement of non-canonical pathways needs to be further investigated. The TGF-beta and SMAD signaling pathway induces EMT and gastric cancer [58], while the PI3K/AKT/mTOR signaling pathway modulates EMT and gastric cancer [59]. Hippo signaling is also implicated in gastric cancer [60]. The pathway network of gastric cancer and other cancers, with cross-talk among various signaling pathways, would be interesting to investigate in the future.

## 4. Conclusions

AOP298, entitled “increase in ROS leading to human treatment-resistant gastric cancer,” illustrates a pathway beginning with chronic increases in ROS, inducing Wnt signaling activation and leading to EMT and treatment-resistant gastric cancer in humans. Its description includes a mechanism of drug resistance, metastasis, and gastric cancer progression, which can be applied to the risk assessment of anti-cancer drugs, such as drug resistance prediction, and the development of anti-cancer treatments.

## Figures and Tables

**Table 1:** AOP298-related information.

Item	Title
AOP	Increase in reactive oxygen species (ROS) leading to treatment-resistant gastric cancer
MIE	KE1115: Increase in reactive oxygen species (ROS)
KE1	KE1754: Porcupine-induced Wnt secretion and Wnt signaling activation
KE2	KE1755: Beta-catenin activation
KE3	KE1457: Epithelial–mesenchymal transition
AO	KE1651: Treatment-resistant gastric cancer

**Table 2:** Support for biological plausibility of KERs in AOP298.

Item	Evidence
MIE => KE1: Increase in ROS leads to Porcupine-induced Wnt secretion and Wnt signaling activation	Biological plausibility of MIE => KE1 is moderate.
	Rationale: Increase in ROS is caused by/causes DNA damage, which will alter several signaling pathways, including Wnt signaling. ROS stimulate inflammatory factor production and Wnt/beta-catenin signaling [24].
KE1 => KE2: Porcupine-induced Wnt secretion and Wnt signaling activation leads to beta-catenin activation	Biological plausibility of KE1 => KE2 is moderate.
	Rationale: Secreted Wnt ligand stimulates Wnt/beta-catenin signaling, where beta-catenin is activated. Wnt ligand binds to frizzled receptor, which leads to GSK3beta inactivation. GSK3beta inactivation leads to beta-catenin dephosphorylation, which avoids the ubiquitination of the beta-catenin and stabilizes beta-catenin [13].
KE2 => KE3: Beta-catenin activation leads to epithelial–mesenchymal transition (EMT)	Biological plausibility of KE2 => KE3 is moderate.
	Rationale: Beta-catenin activation, which includes stabilizing the dephosphorylated beta-catenin and translocation of beta-catenin into the nucleus, induces the formation of the beta-catenin–TCF complex and transcription of transcription factors, such as Snail, Zeb, and Twist [18,25,26,27,28]. EMT-related transcription factors, including Snail, ZEB, and Twist, are up-regulated in cancer cells [29]. Transcription factors such as Snail, ZEB, and Twist bind to the E-cadherin (CDH1) promoter and inhibit CDH1 transcription via the consensus E-boxes (5′-CACCTG-3′ or 5′-CAGGTG-3′), which leads to EMT [29].
KE3 => AO: Epithelial–mesenchymal transition (EMT) leads to treatment-resistant gastric cancer	Biological plausibility of KE3 => AO is moderate.
	Rationale: Some cells exhibiting EMT demonstrate features of cancer stem cells (CSCs) which are related to cancer malignancy [14,30,31,32]. The EMT phenomenon is related to cancer metastasis and cancer therapy resistance [33,34]. Increased expression of enzymes that degrade extracellular matrix components and decrease adhesion to the basement membrane in EMT cause the cell to escape from the basement membrane and induce metastasis [34]. Morphological changes observed during EMT are associated with therapy resistance [34].

**Table 3:** Support for essentiality of KEs in AOP298.

Item	Evidence
MIE: Increase in ROS	Essentiality of the MIE is high.
	Rationale for essentiality of the MIE in the AOP: Increase in ROS contributes to the initiation and development of human gastric cancer [35].
KE1: Porcupine-induced Wnt secretion and Wnt signaling activation	Essentiality of KE1 is moderate.
	Rationale for essentiality of KEs in the AOP: Wnt signaling activation is essential for the subsequent beta-catenin activation and cancer resistance [36].
KE2: Beta-catenin activation	Essentiality of KE2 is moderate.
	Rationale for essentiality of KEs in the AOP: Beta-catenin activation is essential for Wnt-induced cancer resistance [36].
KE3: Epithelial–mesenchymal transition (EMT)	Essentiality of KE3 is moderate.
	Rationale for essentiality of KEs in the AOP: EMT is essential for Wnt-induced cancer promotion and the acquisition of resistance to anti-cancer drugs [6,7,36,37].

**Table 4:** Empirical support for KERs in AOP298.

Item	Evidence
MIE => KE1: Increase in ROS leads to Porcupine-induced Wnt secretion and Wnt signaling activation	Empirical support of MIE => KE1 is moderate.
	Rationale: Production of ROS and DNA double-strand break causes tissue damage [38]. ROS-related signaling induces Wnt/beta-catenin pathway activation [39].
KE1 => KE2: Porcupine-induced Wnt secretion and Wnt signaling activation leads to beta-catenin activation	Empirical support of KE1 => KE2 is moderate.
	Rationale: Disheveled (DVL), a positive regulator of Wnt signaling, forms the complex with frizzled (FZD) and triggers Wnt signaling together with Wnt coreceptor low-density lipoprotein (LDL) receptor-related protein 6 (LRP6) [26,40]. Wnt binds to FZD and activates Wnt signaling [26,41,42]. Wnt binding towards FZD induces the formation of the protein complex with LRP5/6 and DVL, leading to downstream signaling activation, including beta-catenin [13].
KE2 => KE3: Beta-catenin activation leads to epithelial–mesenchymal transition (EMT)	Empirical support of the KE2 => KE3 is moderate.
	Rationale: The inhibition of c-MET, which is overexpressed in diffuse-type gastric cancer, induces increase in phosphorylated beta-catenin and decrease in beta-catenin and Snail [18]. Garcinol, which has an anti-cancer effect, increases phosphorylated beta-catenin, decreases beta-catenin and ZEB1/ZEB2, and inhibits EMT [25]. The inhibition of sortilin by AF38469 (a sortilin inhibitor) or small interference RNA (siRNA) results in a decrease in beta-catenin and Twist expression in human glioblastoma cells [28]. Histone deacetylase inhibitors affect EMT-related transcription factors, including ZEB, Twist, and Snail [43]. Snail and Zeb induce EMT and suppress E-cadherin (CDH1) [29,44,45].
KE3 => AO: Epithelial–mesenchymal transition (EMT) leads to treatment-resistant gastric cancer	Empirical support of KE3 => AO is moderate.
	Rationale: EMT activation induces the expression of multiple members of the ATP-binding cassette (ABC) transporter family, which results in resistance to doxorubicin [14,46]. TGFbeta-1-induced EMT results in the acquisition of cancer stem cell (CSC)-like properties [14,47]. Snail-induced EMT induces cancer metastasis and resistance to dendritic cell-mediated immunotherapy [48]. Zinc finger E-box-binding homeobox (ZEB1)-induced EMT results in the relief of miR-200-mediated repression of programmed cell death 1 ligand (PD-L1) expression, a major inhibitory ligand for the programmed cell death protein (PD-1) immune-checkpoint protein on CD8^+^ cytotoxic T lymphocyte (CTL), and subsequently CD8^+^ T-cell immunosuppression and metastasis [49].

## Data Availability

The information on AOP298 can be downloaded at https://aopwiki.org/aops/298 (accessed on 4 December 2025). This AOP report summarizes the content of AOP298 and is a scientific review part of the OECD AOP project: https://www.oecd.org/en/topics/sub-issues/testing-of-chemicals/adverse-outcome-pathways.html (accessed on 4 December 2025).

## Supplementary Materials

The following supporting information can be downloaded at https://aopwiki.org/aopwiki/snapshot/pdf_file/298-2025-08-15T02:01:48+00:00.pdf (accessed on 4 December 2025), Document S1: PDF snapshot of AOP298 (https://aopwiki.org/aopwiki/snapshot/pdf_file/298-2025-08-15T02:01:48+00:00.pdf) (accessed on 4 December 2025).

## Abbreviations

The following abbreviations are used in this manuscript:

ABC	ATP-binding cassette
AOP	Adverse outcome pathway
AO	Adverse outcome
CDH1	E-cadherin
CSC	Cancer stem cell
CTL	Cytotoxic T lymphocyte
DVL	Disheveled
ROS	Reactive oxygen species
EMT	Epithelial–mesenchymal transition
FZD	Frizzled
IPA	Ingenuity pathway analysis
KE	Key event
KER	Key event relationship
LDL	Low-density lipoprotein
LRP6	LDL receptor-related protein 6
MIE	Molecular initiating event
NADPH	Nicotinamide adenine diphosphate
PD-L1	Programmed cell death 1 ligand
ZEB1	Zinc finger E-box-binding homeobox
